# Bone erosions in rheumatoid arthritis can be repaired through reduction in disease activity with conventional disease-modifying antirheumatic drugs

**DOI:** 10.1186/ar1943

**Published:** 2006-04-28

**Authors:** Haruko Ideguchi, Shigeru Ohno, Hideaki Hattori, Akiko Senuma, Yoshiaki Ishigatsubo

**Affiliations:** 1 Intractable Disease Center, Yokohama City University Medical Center, Yokohama, Japan; 2 Department of Rheumatology, Yokohama Minami Kyousai Hospital, Yokohama, Japan; 3 Department of Internal Medicine and Clinical Immunology, Yokohama City University Graduate School of Medicine, Yokohama, Japan

## Abstract

We conducted the present study to determine whether repair of erosions occurs in patients with rheumatoid arthritis (RA) treated with conventional disease-modifying anti-rheumatic drugs (DMARDs) and to compare clinical characteristics between patients exhibiting and not exhibiting erosion repair. We included in the study a total of 122 RA patients who fulfilled the 1987 American College of Rheumatology criteria for RA; all patients had paired sequential radiographs of both hands and wrists showing erosive changes at baseline. Patients were classified into two groups according to the presence of repair of erosions at follow up, namely the 'repair observed' and 'repair not observed' groups. Clinical characteristics, disease activity, radiographic scores and treatment in the two groups were compared. Forty-four repairs were observed in 13 patients (10.7%). Compared with the repair not observed group, the functional class of the patients in the repair observed group was lower at baseline (*P *< 0.01) and the mean disease activity was lower at follow up (*P *< 0.005). The changes in radiographic scores per year (total radiographic score and erosion score) were lower (*P *< 0.05 and *P *< 0.01, respectively) in the repair observed group. No difference in treatment was observed. Repair of erosions was detected in 10.7% of RA patients treated with conventional DMARDs. Repairs were associated with low functional class at baseline and low disease activity at follow up. These observations support the importance of reduction in disease activity in RA patients. Because repair of erosions was detected in a substantial number of patients, assessment of erosion repair should be incorporated into the radiographic evaluation and scoring of RA.

## Introduction

Rheumatoid arthritis (RA) is a chronic, destructive autoimmune inflammatory disorder of unknown aetiology that occurs in about 1% of the adult population [1].

The radiograph has evolved into the 'gold standard' for evaluation of RA progression because it best demonstrates the anatomical destruction of joint structures [2]. However, repair of erosions or reparative changes in RA have rarely been reported [3-10]. There are several possible reasons for this. First, radiographs are rarely obtained in patients who appear to be experiencing remission, in whom repair phenomena may be observed. Second, most clinical trials are conducted in patients with longstanding destructive RA with high disease activity, and in such cases it is difficult to define repair clearly. Third, there is an interval between clinical findings and corresponding radiographic phenomena in clinical trials, and most clinical trials have insufficient follow up to identify repair phenomena. Fourth, the most commonly used scoring methods, namely those of Sharp and Larsen and their groups, are not designed to describe reparative changes. The Sharp/van der Heijde method includes 16 areas for erosions and 15 for joint space narrowing in each hand. The erosion score per joint can range from 0 to 5. Joint space narrowing is scored with range from 0 to 4. The maximum erosion score of all joints in both hands is 160 and the maximum score for joint space narrowing in all joints of both hands is 120. Finally, serial high-quality radiographs must be obtained to permit detection of repair.

In recent years considerable advances have been made in the pharmacological management of RA [11]. Previously, the mainstays of treatment for RA were disease-modifying antirheumatic drugs (DMARDs), which suppress the inflammatory process and slow disease progression. Today's biological agents are specifically designed to block key mediators of the RA inflammatory process.

Recent trials with tumour necrosis factor-α blocking drugs in RA have demonstrated improvements in radiographic scores, suggesting drug-induced repair of erosions [12-14]. These findings have renewed interest in the radiographic detection of repair, and Sharp and coworkers [15] instituted a subcommittee of the OMERACT (Outcome Measures in Rheumatology) Imaging Committee in a first attempt to confirm whether repair occurs in RA and, if so, to determine how repair should be assessed. They provided evidence that repair of bone damage does occur in RA, resulting in some degree of improvement, which was recognized by the majority of a panel of experts. A consensus on definitions for the morphological features of repair was achieved for the following: sclerosis, cortication, filling-in, remodeling and restoration [15].

We wondered whether repair of erosions can be detected in the real world setting among RA patients who have longstanding arthritis, who do not achieve remission easily. To examine whether repair of erosions in RA patients treated with conventional DMARDs can be detected, we examined 122 RA patients with paired sequential radiographs of both hands and wrists. We also assessed the clinical characteristics of these patients.

## Materials and methods

### Patients

All patients were diagnosed with RA according to the 1987 American College of Rheumatology criteria for RA. From among RA patients seen in the rheumatology clinic by two of the authors (HI and SO) at Yokohama City University Medical Center between January 2001 and March 2004, patients with paired sequential radiographs of both hands and wrists were enrolled in this study.

### Clinical evaluation

Clinical characteristics, including patient's sex, age, disease duration, rheumatoid factor status, prescription (dose of prednisone, bisphosphonates and DMARDs such as methotrexate and sulfasalazine), functional class (determined using American College of Rheumatology criteria) and RA stage (determined according to Steinbrocker criteria), were recorded.

Disease activity was assessed using the Disease Activity Score (DAS) in 28 joints, DAS28-3. Modifications to the original DAS index have been applied, yielding the DAS28 (which is a composite index that includes variables such as the number of tender and swollen joints using 28 joint counts, erythrocyte sedimentation rate (ESR), and the patients' assessment of disease activity) and DAS28-3 (excluding patients' assessment of disease activity from DAS28) indices; these indices have been and validated and, for reasons of simplicity, are preferred over the original index in clinical practice [16-18]. The DAS28-3 value was calculated as follows:

DAS28-3 score = (0.56 ×  + 0.28 ×  + 0.7 × ln[erythrocyte sedimentation rate]) × 1.08 + 0.16.

### Radiographic evaluation

#### Detection of repair of erosions

Standard radiographs of the hands and wrists were obtained in two planes (anteroposterior and oblique projections). Radiographic examinations were interpreted independently by two board-certified rheumatologists with musculoskeletal reading experience (HI and SO). The films were reviewed to identify erosion repair specifically. Bone erosion was defined as a discrete interruption of the cortical surface, based on standard plain film radiograph criteria [19,20]. The films were evaluated with known sequences in order to ensure maximum sensitivity in detecting erosion repair [21,22]. The evaluators were blinded to patient identity. Repair was defined as follows [10]: category 1, reappearance of the cortical plate at a bone site where it had been destroyed; category 2, partial or complete filling in of an erosion; and category 3, subchondral bone sclerosis and osteophyte formation (secondary osteoarthritis). Where there was a difference in opinion regarding whether repair was present, these cases were discussed until agreement was reached. To confirm the existence of repair, these films were re-examined in random order, without knowledge of the sequence, by two other rheumatologists (HH and AS) as well as by the initial ones.

#### Radiographic scoring method

Radiographic joint damage of the hands and wrists was assessed using the van der Heijde modification to the total Sharp scoring system (vdH-S) [23]. Two rheumatologists (HI and SO) scored the films of each patient independently, with knowledge of the order of the radiographs. Erosion scores can range from 0 to 160. Joint space narrowing (JSN) scores can range from 0 to 120. Total radiographic scores can range from 0 to 280. Readers were allowed to record improvement in scores. For each set of radiographs, the mean score by the two readers was used in the analysis. In the present study the smallest detectable change was 12.8, which reflects that component of a measurement that was statistically attributable to error from the measurement process [24-26]. Because the time interval between pairs of radiographs varied among patients, radiographic progression was determined by dividing the difference between scores of each pair of radiographs by the years that had elapsed between radiographs; the yearly adjusted progression rate for each patient was expressed as changes in score (total radiographic, erosion, or JSN) per year.

### Statistical analysis

Statistical analysis was conducted using SPSS 11.0 statistical analysis software (SPSS Inc., Chicago, Illinois, USA). We analyzed group differences with respect to sex, rheumatoid factor and prescribed bisphosphonates using Fisher's exact probability test. Then, we examined whether the data exhibited a normal distribution using F test, and in cases of normal distribution we used parametric examination. If equal variance was gained, we used the Student's *t *test. We analyzed group differences in age and DAS28-3 score using Student's *t *test. If equal variance was not gained, then we used the Mann-Whitney *U *test. We also analyzed group differences in disease duration, dosage of prednisone, class, radiographic score and prescribed DMARDs using the Mann-Whitney *U *test. Statistical significance was defined as a *P *value less than 0.05. Significant variables in univariate analysis were entered simultaneously into multivariate logistic regression to identify variables with independent predictive value for repair.

### Ethics

Informed consent was obtained from each patient, and the Ethics Committee of the Yokohama City Medical Center approved the study protocol.

## Results

RA patients with paired sequential radiographs of both hands and wrists exhibiting erosive changes at baseline were included in the study. A total of 122 patients (103 females [84.4%] and 19 males [15.6%], aged 22–85 years) were enrolled (Table 1).

Forty-four repairs were detected in 13 patients (10.7%). Of these repairs, initial difference in opinion between HI and SO existed for only one repair. Subsequently, to confirm the existence of repair, 44 pairs of images were independently presented to two other readers as well as HI and SO, without knowledge of the sequence, and they were asked to indicate which image was worse on global evaluation of erosion and which erosion was larger in size. Complete agreement for both evaluations among the four readers was achieved in 95.5% of repairs. Based on this high level of interobserver agreement, these 44 repairs in 13 patients were included in the following analysis. According to the definition of repair given above, the 44 repairs were classified as follows: five repairs were deemed to be in category 1 (reappearance of the cortical plate at a bone site where it had been destroyed); 39 repairs were in category 2 (partial or complete filling in of an erosion); and no repair was in category 3 (subchondral bone sclerosis and osteophyte formation). In other words, none of the erosions was judged as exhibiting 'repair' as a result of osteophyte development.

**Figure 1:**
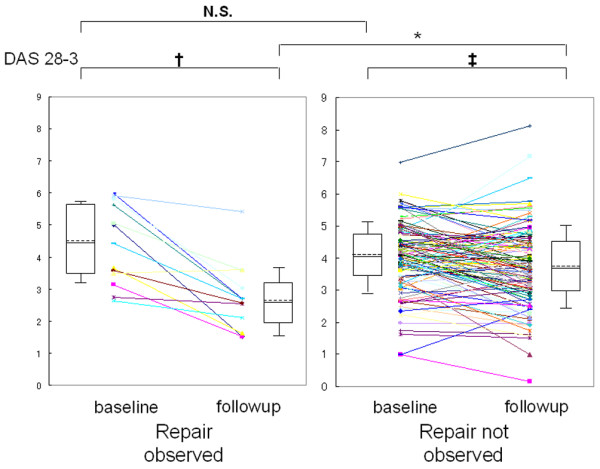
Analysis of DAS28-3 values. The DAS28-3 values at baseline and follow up are shown for the 'repair observed' and 'repair not observed' groups. For the repair observed group the mean ± standard deviation (solid horizontal lines and bars) and median (interquartile range [35]; dashed horizontal lines and boxed areas) DAS28-3 values at baseline were 4.4 ± 1.2 and 4.4 (3.4–5.7), respectively; at follow up they were 2.7 ± 1.1 and 2.7 (2.0–3.2). For the repair not observed group the mean ± standard deviation and median (interquartile range) DAS28-3 values at baseline were 4.0 ± 1.1 and 4.1 (3.4–4.7), respectively; at followup they were 3.7 ± 1.2 and 3.7 (2.9–4.5). *P < 0.005, ^†^*P *< 0.001, ^‡^*P *< 0.005 (determined by the Student's *t *test). DAS, Disease Activity Score.

There were no significant differences between the groups of those exhibiting repair (for instance, the 'repair observed' group) and those in whom there was no evidence of repair (for instance, the ' repair not observed' group) for any of the following characteristics: including demographic variables (sex, age and disease duration), rheumatoid factor positivity, dosage of prednisone, prescribed bisphosphonates, radiographic score in vdH-S (total radiographic, erosion and JSN score; Table 2) and DAS28-3 score at baseline (Figure 1). Class status at baseline was significantly lower in the repair observed group (*P *< 0.01). DAS28-3 score at follow up was significantly lower in the repair observed group (*P *< 0.005; Figure 1), as was the change in DAS28-3 score (ΔDAS28-3; *P *< 0.001) and ΔDAS28-3/year (*P *< 0.01). These data suggest that repair of erosions can be achieved in patients with low disease activity at follow up and/or patients exhibiting good response to treatment.

Prescribed DMARDs are summarized in Table 3. No significant differences in prescriptions of prednisone and bisphosphonates were observed between the two groups.

**Figure 2:**
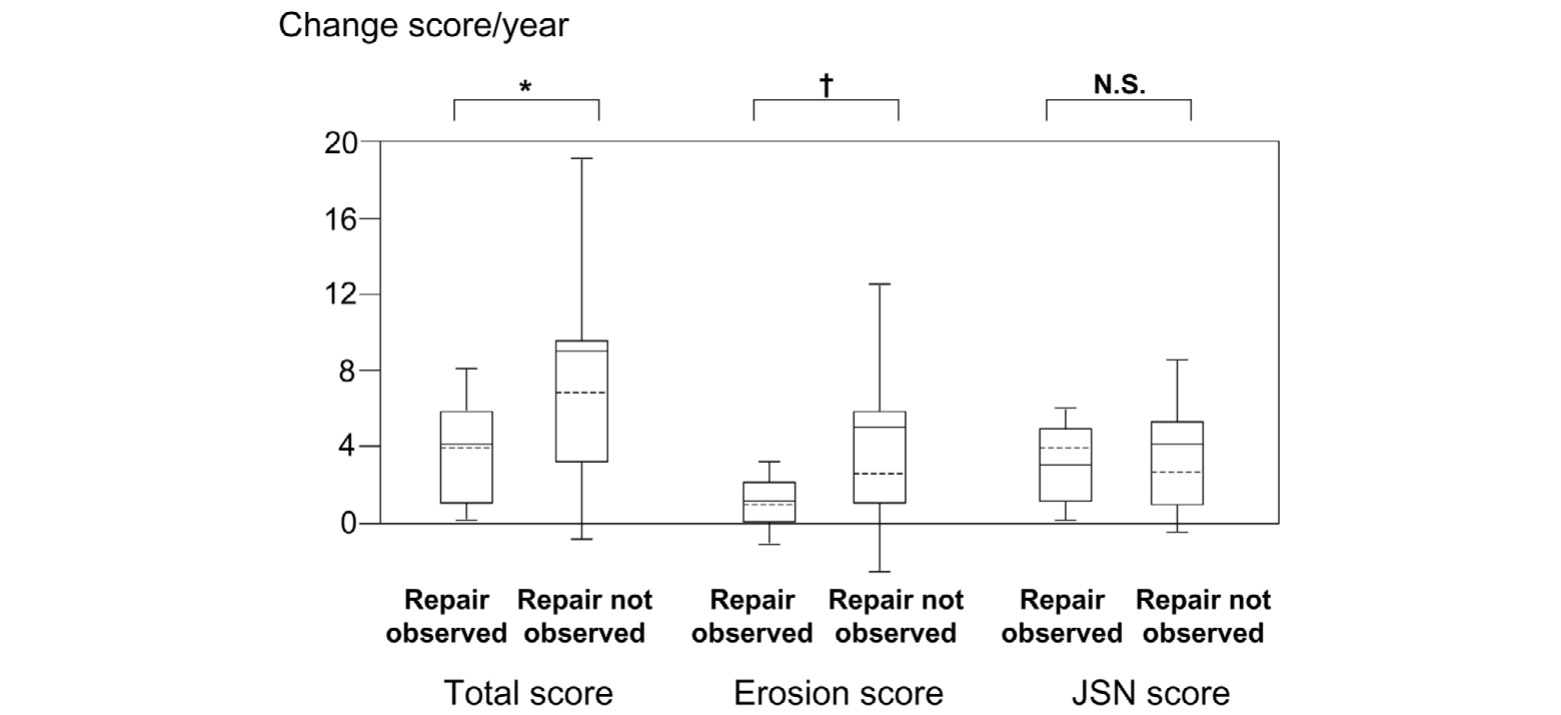
Differences in the radiographic progression in vdH-S. The mean ± standard deviation (solid horizontal lines and bars) and median (interquartile range [35]; dashed horizontal lines and boxed areas) change in total radiographic score/year were 4.1 ± 4.0 and 4.0 (1.1–6.0) in the repair observed group, respectively; they were 9.3 ± 10.0 and 7.0 (3.5–9.9) in the repair not observed group (*P < 0.05). The mean ± standard deviation and median (interquartile range) change in erosion score/year were 1.1 ± 2.2 and 1.0 (0.0–2.3) in the repair observed group, respectively; they were 5.2 ± 7.6 and 2.7 (1.1–6.1) in the repair not observed group (^†^*P *< 0.01). The mean ± standard deviation and median (interquartile range) change in JSN score/year were 3.1 ± 3.0 and 3.9 (1.2–5.0) in the repair observed group, respectively; they were 4.1 ± 4.4 and 3.0 (1.0–5.6) in the repair not observed group (not significant).

**Figure 3:**
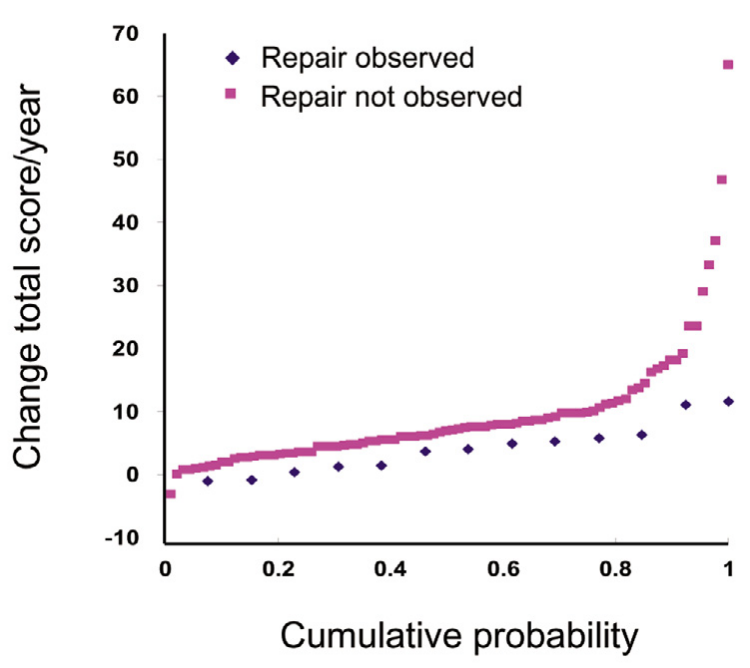
Probability plot: change/year in total radiographic score.

**Figure 4:**
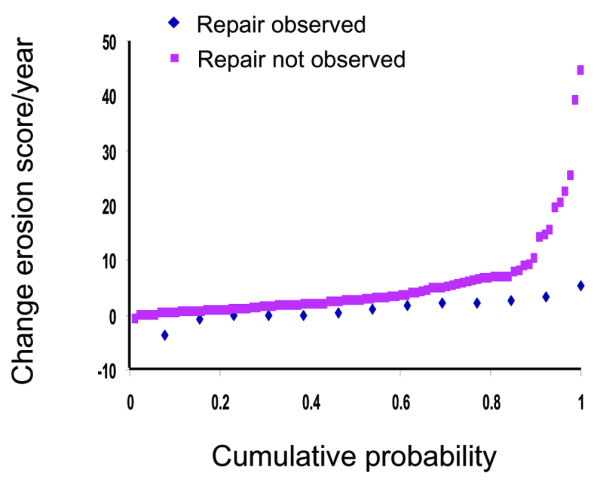
Probability plot: change/year in erosion score.

**Figure 5:**
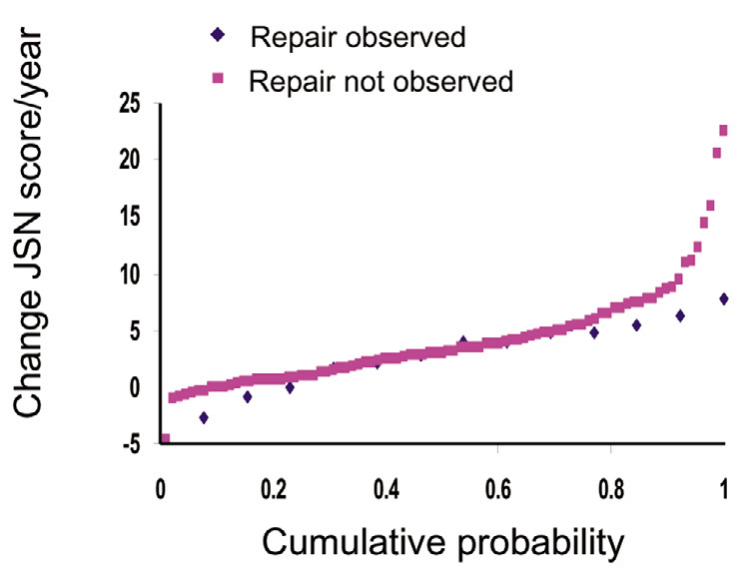
Probability plot: change/year in JSN score. JSN, joint space narrowing.

There were no significant differences between groups in total radiographic score, erosion score, or JSN score at baseline. Differences in radiographic progression between the two groups are illustrated in Figure 2. Although there was no difference in radiographic score at baseline, changes in the total radiographic score and erosion score at follow up were significantly lower in the repair observed group. The changes in total radiographic score/year were 4.1 ± 4.0 in the repair observed group and 9.3 ± 10.0 in the repair not observed group (*P *< 0.05). The changes in erosion score/year were 1.1 ± 2.2 in the repair observed group and 5.2 ± 7.6 in the repair not observed group (*P *< 0.01). The changes in JSN score/year were 3.1 ± 3.0 in the repair observed group and 4.1 ± 4.4 in the repair not observed group (not significant). The differences between groups in changes in scores/year (from follow up to baseline) are illustrated as probability plots in Figures 3, 4, 5.

Univariate analysis revealed that class status at baseline (*P *< 0.01), DAS28-3 at follow up (*P *< 0.005), ΔDAS28-3 (*P *< 0.001), ΔDAS28-3/year (*P *< 0.01), change in total radiographic score/year (*P *< 0.05) and change in erosion score/year (*P *< 0.01) were significant predictors of repair. These factors were entered into a multivariate logistic regression model to identify variables with independent predictive value for repair. ΔDAS28-3 was found to be a significant independent predictor of repair (*P *< 0.007).

**Figure 6:**
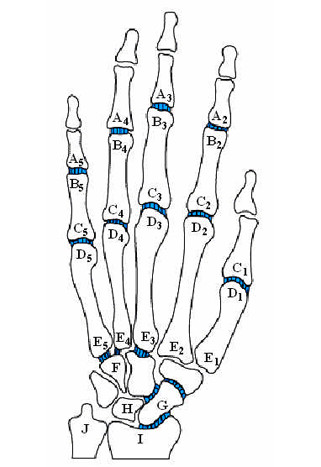
Diagram of the left hand showing sites where erosion repair was observed. The dorsal aspect of the left hand is shown: A = base of middle phalanx, B = head of proximal phalanx, C = base of proximal phalanx, D = head of metacarpal bone, E = base of metacarpal bone, F = hamate bone, G = scaphoid bone, H = lunate bone, I = distal radius, and J = distal ulna.

Bone sites of erosion repair are summarized in Table 4. The dorsal bone aspect of the left hand is shown in Figure 6. A total of 44 repairs were detected at 10 bone sites in 13 patients. Twenty repairs (45.5%) were observed in proximal interphalangeal (PIP) joints.

**Figure 7:**
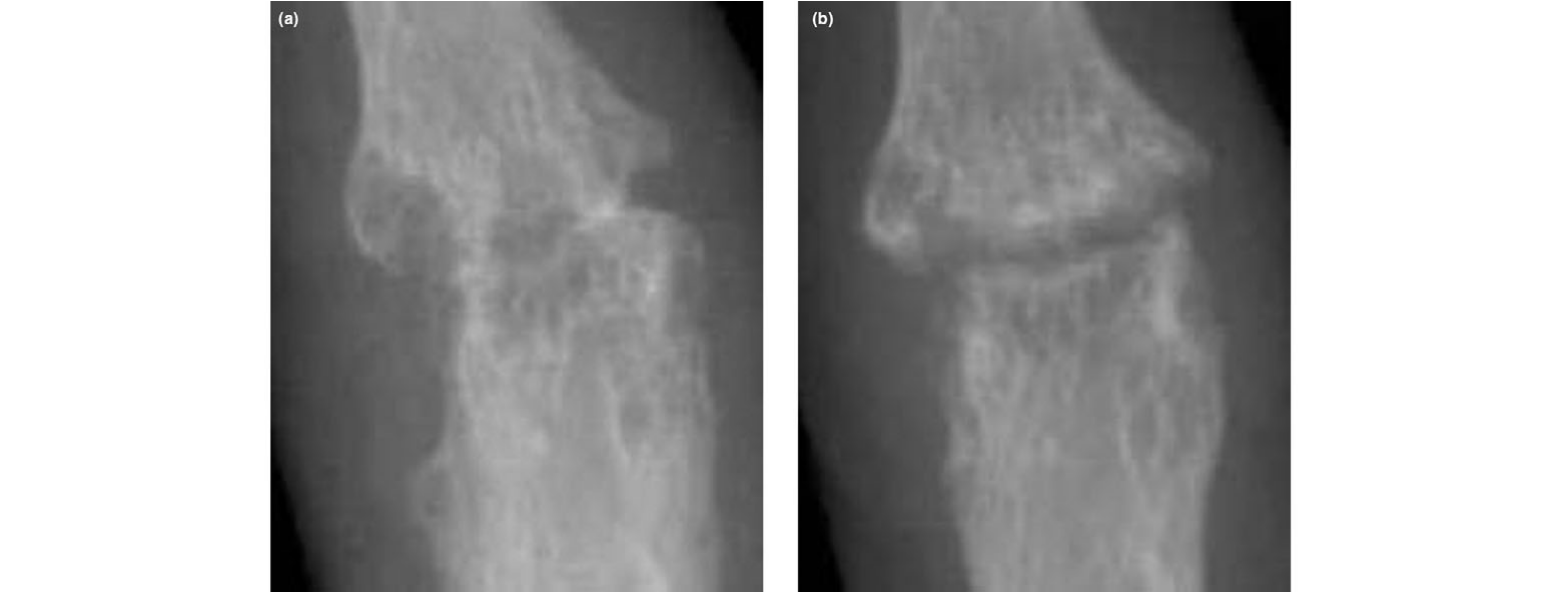
Recortication and filling in at a right fourth PIP joint. Images of the same hand are shown from **(a) **October 2002 and **(b) **November 2003. PIP, proximal interphalangeal.

Representative images showing repair of erosions are given in Figure 7.

## Discussion

Most of the scoring systems of radiographic data and their multiple modifications are designed to quantify the speed of progressive destruction over time in selected joints of the hands, wrists and feet. However, they are not designed to document improvement. If readers were allowed to score improvements in scores, these scoring methods – with appropriate statistical analysis – may accurately reflect repair in serial films. However, in reality the majority of healing phenomena do not result in changes to radiographic scores [10].

Several investigations have reported that magnetic resonance imaging (MRI) and ultrasonography are highly sensitive in detecting erosions in the hands and wrists of patients with RA [27-29]. Indeed, erosions shown by MRI may be visible 6–12 months or more before they are observed on radiographs, providing a means of early detection and immediate therapeutic intervention [28,30]. Stewart and coworkers [31] examined 41 patients with RA. MRI scans of the dominant wrist in 31 patients obtained at 1 year and 6 years were compared. Twenty-two patients had an increase in erosion score in the interval and three patients exhibited a decrease in erosion score, suggesting erosion repair.

Based on these findings, we investigated whether repair of erosions can be detected in RA patients treated with conventional DMARDs at our rheumatology clinic, and we compared clinical characteristics between patients exhibiting and not exhibiting erosion repair. To ensure optimal sensitivity in detecting erosion repair, the films were evaluated with knowledge of the sequence. Although this is an issue that still attracts much debate, one study [32] suggested that films should preferably be read in chronological order because this leads to an increase in the detection of clinically relevant changes, without serious overestimation of irrelevant changes. In another study [33] reading films in chronological order was shown to yield a better signal-to-noise ratio when compared with paired reading. We chose the chronological approach in the present study because we believe it to be the most sensitive scoring method; however, more data are needed before we may arrive at a definitive conclusion regarding the optimal approach in observational studies. Furthermore, interobserver agreement was tested independently by a total of four rheumatologists without knowledge of sequence, resulting in high agreement.

We assessed radiographic joint damage using vdH-S. There were no significant differences between groups in total radiographic score, erosion score, or JSN score at baseline. The change in total radiographic score/year (*P *< 0.05) and change in erosion score/year (*P *< 0.01) were significantly lower in the repair observed group. To our surprise, patients with erosion repair at any bone site of the hands exhibited lower overall radiographic progression rates, as evaluated using vdH-S, which does not include scoring of healing phenomena. The change in erosion score/year of 1.1 ± 2.2 in the repair observed group means that erosive progression does occur simultaneously with repair in other joints in the same patient. In fact, new erosions were observed in four patients (30.8%) in the repair observed group. Another interesting finding from our study was that erosion repair was observed in patients who were not taking any DMARDs. This suggests the existence of a physiological process of self-repair in RA patients with low disease activity.

The great majority of repairs (20 repairs [45.5%]) were detected in PIP joints. We believe we can partially explain this by the fact that more erosions were observed at PIP joints than at other bone sites at baseline. The number of 'repairs' becomes higher with greater numbers of erosions at baseline.

The process of bone destruction in RA is considered to be correlated with arthritis activity [34], although evidence is accumulating that bone destruction can occur independent of arthritis activity [35-38]. Molenaar and coworkers [35] followed up 187 patients with RA who were in remission clinically and radiologically for 2 years. After 2 years of follow up, remission persisted in 52% of patients. Clinically relevant progression of damage was more frequent in patients with exacerbation than in those with persistent remission.

However, in 15% of patients with persistent remission, erosions developed in previously unaffected joints. Those investigators concluded that the DAS area under the curve was a stronger predictor of radiographic progression than were baseline variables and absence of persistent remission. In the present study there were eight patients in persistent remission, defined as DAS28-3 score below 2.6. Erosion repair was detected in none of these patients; indeed, three patients (37.5%) even exhibited radiographic progression exceeding the smallest detectable change (total radiographic score increase in vdH-S >12.8). On the other hand, although 12 patients (92.3%) in the repair observed group exhibited reduced DAS28-3 score at follow up, one patient actually had a slight increase of disease activity (0.1 increases in ΔDAS28-3). In the repair observed group, 10 patients were in remission or had low disease activity at follow up (DAS28-3 score <3.2), two patients had intermediate activity (DAS28-3 score greater than 3.2 but less than 5.1) and one patient had high disease activity (DAS28-3 score >5.1). Our data confirm the finding of Molenaar and coworkers [35] that bone destruction can occur independently of clinical activity.

The present study has several limitations resulting from its retrospective design. The number of patients with erosion repair was relatively small, and the radiographic evaluations were limited to hands because the number of radiographs of the feet taken at routine clinical setting was small. However, these limitations do not diminish the importance of the study, which, to our knowledge, is the first to explore the clinical characteristics of RA patients exhibiting erosion repair.

## Conclusion

Our study suggests that structural repair can be achieved by reducing disease activity in RA, and this phenomenon is not limited to patients with early disease or treatment with biological agents. The importance of targeting remission with aggressive therapy in all patients with RA is highlighted. A reliable method for evaluating and scoring of erosion repair must be established and factors predictive of bone repair identified in the near future.

## Abbreviations

DAS = Disease Activity Score; DMARD = disease-modifying antirheumatic drugs; JSN = joint space narrowing; MRI = magnetic resonance imaging; PIP = proximal interphalangeal; RA = rheumatoid arthritis; vdH-S = the van der Heijde modification of the total Sharp scoring system.

## Competing interests

The authors declare that they have no competing interests.

## Authors' contributions

HI and SO designed and organized the study. HI performed the statistical analysis. HI, SO, HH and AS were involved in the radiographic examinations. HI, SO and YI were involved in writing the report. All authors read and approved the final manuscript.

## Figures and Tables

**Table 1:** Characteristics of the study population

Characteristic	Group		*P *value
		
	Repair observed	Repair not observed	
Patients (*n*)	13	109	
Female (*n *[%])	10 (76.9%)	93 (85.3%)	NS^a^
Age (mean [range]; years)	64.2 ± 9.8 (39–78)	60.6 ± 12.6 (22–85)	NS^b^
Disease duration (mean [range]; years)	12.5 ± 9.6 (2.7–38)	14.3 ± 10.8 (1–45)	NS^c^
RF positivity (*n *[%])	11(84.6%)	85(78.0%)	NS^a^
Dosage of prednisone (mean [range]; mg)	2.0 ± 2.1 (0.0–6.0)	3.7 ± 3.3 (0.0–14.5)	NS^c^
Prescribed bisphosphonate (*n *[%])	8 (61.5%)	79 (72.5%)	NS^a^
Functional class			
I	4	10	<0.01^c^
II	9	78	
III	0	18	
IV	0	3	
RA stage			
I	0	0	NS^c^
II	4	36	
III	2	19	
IV	7	54	
DAS28-3 score at baseline (mean [range])	4.4 ± 1.2 (2.7–6.0)	4.0 ± 1.1 (1.0–7.0)	NS^b^
DAS28-3 score at follow up (mean [range])	2.7 ± 1.1 (1.5–5.4)	3.7 ± 1.2 (0.2–8.1)	<0.005^b^
ΔDAS28-3 (mean [range])	-1.7 ± 1.2 (-3.5 to +0.1)	-0.3 ± 1.0 (-2.4 to +2.6)	<0.001^b^
Time interval between pairs of radiographs, years (mean [range])	2.3 ± 1.0 (1.4–3.1)	1.6 ± 1.8 (1.0–1.6)	NS^b^
ΔDAS28-3/year (mean [range])	-0.9 ± 0.8 (-2.6 to +0.1)	-0.2 ± 0.9 (-2.8 to +3.4)	<0.01^b^

RA functional status was determined using American College of Rheumatology criteria, and RA stage was determined using Steinbrocker criteria. ^a^Fisher's exact probability test; ^b^Student's *t *test; ^c^Mann-Whitney *U *test. DAS, Disease Activity Score; RF, rheumatoid factor.

**Table 2:** Radiographic joint damage scores at baseline

Score	Group	
	
	Repair observed	Repair not observed
Total radiographic score^a^		
Mean ± SD	117.1 ± 39.2	106.1 ± 66.3
Median (range)	119.0 (47.0–188.5)	100.5 (9.0–242.0)
Interquartile range	86.3–138.1	48.0–153.8
*P*^b^		NS
Erosion score^c^		
Mean ± SD	54.7 ± 26.7	42.6 ± 38.3
Median (range)	57.5 (9.5, 101.0)	28.8 (0.5, 135.0)
Interquartile range	30.1, 69.0	11.5, 66.8
*P*^b^		NS
JSN score^d^		
Mean ± SD	62.3 ± 13.9	63.6 ± 30.9
Median (range)	62.5 (37.5, 87.5)	67.3 (8.5, 115.0)
Interquartile range	55.5, 72.0	34.0, 92.8
*P*^b^		NS

^a^Scores can range from 0 to 280, with higher scores indicating more joint damage. ^b^Mann-Whitney *U *test. ^c^Scores can range from 0 to 160. ^d^Scores can range from 0 to 120. JSN, joint space narrowing; NS, not significant; SD, standard deviation.

**Table 3:** Prescriptions of DMARDs, prednisone and bisphosphonates

Drug	Repair observed	Repair not observed
DMARDs		
Methotrexate	7 (53.8%)	59 (54.1%)
Sulfasalazine	4 (30.8%)	35 (32.1%)
Methotrexate and sulfasalazine	1 (7.7%)	18 (16.5%)
Others	1 (7.7%)	11 (10.1%)
None	2 (15.4%)	22 (20.2%)
Prednisone	7 (53.8%)	75 (68.8%)
Bisphosphonates	8 (61.5%)	79 (72.5%)

Values are expressed as *n *(%). DMARDs, disease-modifying antirheumatic drugs.

**Table 4:** Bone sites where repairs of erosions were observed in individual patients

Patient number	Bone site and number of erosions	Number of repairs
1	rB_2 _× 1	1
2	rA_3 _× 2, rB_3 _× 2	4
3	rA_4 _× 1, lA_3 _× 1, lB_3 _× 1	3
4	rC_2 _× 1, lI × 1	2
5	lE_5 _× 1, lF × 1, lG × 1, rH × 1, lH × 1	5
6	rD_2 _× 1, rI × 1	2
7	lA_4 _× 1, lB_4 _× 1, rE_4 _× 1, rE_5 _× 1, rG × 1	5
8	lC_3 _× 1, rE_5 _× 1	2
9	rD_2 _× 1, lH × 1, rI × 1, lI × 1, lJ × 1	5
10	rD_2 _× 1, rI × 1	2
11	rA_5 _× 2, rB_5 _× 2, lI × 1	5
12	rE_5 _× 1, rF × 1	2
13	rA_4 _× 1, lA_5 _× 2, rB_4 _× 1, lB_5 _× 2	6
Total		44

An example of the notation used in the table is as follows: '*r*A_3 _× 2' means two repairs in the base of right third middle phalanx. A, base of middle phalanx; B, head of proximal phalanx; C, base of proximal phalanx; D, head of metacarpal bone; E, base of metacarpal bone; F, hamate bone; G, scaphoid bone; H, lunate bone; I, distal radius; J, distal ulna; l, left hand; r, right hand.
